# Delineation of Subregions in the Early Postnatal Human Cerebellum for Design-Based Stereologic Studies

**DOI:** 10.3389/fnana.2017.00134

**Published:** 2018-01-08

**Authors:** Anna Fichtl, Andreas Büttner, Patrick R. Hof, Christoph Schmitz, Maren C. Kiessling

**Affiliations:** ^1^Chair of Neuroanatomy, Faculty of Medicine, Institute of Anatomy, Ludwig-Maximilians-Universität München, Munich, Germany; ^2^Institute of Forensic Medicine, University of Rostock, Rostock, Germany; ^3^Fishberg Department of Neuroscience and Friedman Brain Institute, Icahn School of Medicine at Mount Sinai, New York, NY, United States

**Keywords:** cerebellum, design-based stereology, humans, postnatal, reproducibility of results, subregions

## Abstract

Recent design-based stereologic studies have shown that the early postnatal (<1 year of age) human cerebellum is characterized by very high plasticity and may thus be very sensitive to external and internal influences during the first year of life. A potential weakness of these studies is that they were not separately performed on functionally relevant subregions of the cerebellum, as was the case in a few design-based stereologic studies on the adult human cerebellum. The aim of the present study was to assess whether it is possible to identify unequivocally the primary, superior posterior, horizontal, ansoparamedian, and posterolateral fissures in the early postnatal human cerebellum, based on which functionally relevant subregions could be delineated. This was tested in 20 human post mortem cerebellar halves from subjects aged between 1 day and 11 months by means of a combined macroscopic and microscopic approach. We found that the superior posterior, horizontal, and posterolateral fissures can be reliably identified on all of the specimens. However, reliable and reproducible identification of the primary and ansoparamedian fissures was not possible. Accordingly, it appears feasible to perform subregion-specific investigations in the early postnatal human cerebellum when the identification of subregions is restricted to crus I (bordered by the superior posterior and horizontal fissures) and the flocculus (bordered by the posterolateral fissure). As such, it is recommended to define the entire cerebellar cortex as the region of interest in design-based stereologic studies on the early postnatal human cerebellum to guarantee reproducibility of results.

## Introduction

The cerebellum is connected to the cerebrum, the brainstem, and the spinal cord by several fiber pathways (e.g., Paxinos, 1990; Roostaei et al., 2014; Witter and De Zeeuw, 2015). It is critically involved in motor and sensory function as well as higher cognitive and emotional functions that can be assigned to different subregions of the cerebellum (e.g., Stoodley and Schmahmann, 2009; Buckner, 2013; Witter and De Zeeuw, 2015). The human cerebellum can be examined at different levels. For example, its function can be studied *in vivo* using positron emission tomography (PET; e.g., Petacchi et al., 2010) and functional magnetic resonance imaging (fMRI; e.g., Schraa-Tam et al., 2012). Structural studies comprise MRI *in vivo* (Schmahmann et al., 1999) and various approaches to understand its microscopic anatomy. In this context, Stoodley and Schmahmann (2009) performed a meta-analysis of more than 50 functional neuroimaging studies of the human cerebellum and found the following: (i) sensorimotor tasks activate the anterior lobe (lobule V) and adjacent lobule VI, with additional foci in lobule VIII; (ii) motor activation is found in lobule VIIIA/B, while somatosensory activation is confined to lobule VIIIB; (iii) the posterior lobe is involved in higher-level tasks; (iv) lobule VI and Crus I are involved in language and verbal working memory, lobule VI in spatial tasks, lobules VI, Crus I and VIIB in executive functions, and lobules VI, Crus I and medial lobule VII in emotional processing; (v) language is heavily right-lateralized and spatial tasks left-lateralized, reflecting crossed cerebro-cerebellar projections; and (vi) emotional processing involves vermal lobule VII, implicated in cerebellar-limbic circuitry. Furthermore, language and executive tasks activate regions of Crus I and lobule VII proposed to be involved in prefronto-cerebellar loops.

Several studies have shown that quantitative-histologic investigations using design-based stereology provide more reliable insight into the normal and pathologic microscopic structure of the human cerebellum than related studies that were not performed with these techniques. In this regard it is of note that Sparks and Hunsaker (2002) hypothesized that the cerebellum plays an important role in the pathogenesis of sudden infant death syndrome (SIDS). This hypothesis is related to the role of the cerebellum in respiratory and cardiovascular control (Cruz-Sánchez et al., 1997; Harper et al., 2000), as well as the hypothesis that in SIDS affected children may suffer from prolonged apnea and suddenly stop breathing (Steinschneider, 1972; Guilleminault et al., 1975). Most probably Gadsdon and Emery (1976) first called attention to the possible involvement of the cerebellum in SIDS. In the following years, additional post mortem studies on the cerebellum of SIDS patients were published, yielding conflicting results. Some of these studies reported no differences between SIDS and control cases (Oehmichen et al., 1989; Riedel et al., 1989). Other studies proposed a developmental delay of the cerebellum in SIDS (Cruz-Sánchez et al., 1997) or reported several changes in the cerebellar cortex in SIDS (Lavezzi et al., 2006, 2007). However, none of these studies were performed using a rigorous design-based stereologic approach. This was performed more recently by Kiessling et al. (2013) who found no alterations in mean total numbers of Purkinje cells and granule cells in the cerebellum of SIDS patients and age- and sex-matched controls. Moreover, using the design-based stereologic probe “space balls” (Calhoun and Mouton, 2000; Mouton et al., 2002), Müller-Starck et al. (2014) found no differences either in mean microvessel length density in the cerebellar layers between the same SIDS cases and controls investigated by Kiessling et al. (2013) or between controls with a low likelihood of hypoxia and those with a higher likelihood of hypoxia. These data did not support the hypothesis of hypoxia in the cerebellum in SIDS.

A potential weakness of the studies by Kiessling et al. (2013) and Müller-Starck et al. (2014) is that they were not performed in functionally relevant subregions in the cerebellum (see Stoodley and Schmahmann, 2009), as had been done in a few other studies applying design-based stereology. For instance, Andersen et al. (2003) found an age-related neuron loss in the human cerebellum starting at ~65 years of age when investigating post mortem brains from subjects without neurological disorders aged between 19 and 84 years. By determining total neuron numbers, these authors investigated four different cerebellar subregions. The greatest neuron loss was reported in the anterior lobe, namely a loss of 40.6% of Purkinje cells and granule cells. In contrast, neuron loss in the posterior lobe, vermis, and flocculonodular lobe was not as remarkable. As a result, Andersen et al. (2003) reported an overall age-related decrease by 11.7% in the total number of Purkinje cells and 12.7% in the total number of granule cells in the human cerebellum. This study demonstrated that design-based stereologic investigations focusing on cerebellar subregions may in fact come to different conclusions than studies performed on the entire human cerebellum.

Accordingly, it appears attractive to study quantitative parameters such as total numbers of cells and microvessel length densities in a subregion-specific manner in SIDS and neurodevelopmental disorders affecting the cerebellum (e.g., Steinlin, 2008; Stoodley, 2016; Stoodley and Limperopoulos, 2016) using design-based stereology. However, the latter would require unequivocal and reproducible identification of subregions that can serve as regions of interest (ROIs) in such studies. In this regard, it is critical to note that the human cerebellum has a much higher structural and functional plasticity during the first year of life than previously thought (Kiessling et al., 2014), and may respond very sensitively to internal and external influences during this time. Specifically, ~85% of the cerebellar granule cells are generated postnatally in humans, and the mean number of granule cells per Purkinje cell in the human cerebellum increases from approximately 480 in the first postnatal month to ~2,700 in the 11th month of life (Kiessling et al., 2014). These data may have important implications for several neuropsychiatric conditions in which cerebellar involvement has been demonstrated, including its potential role in autism (Palmen et al., 2004; Fatemi et al., 2012), autistic characteristics associated with changes of the cerebellar vermis (Hashimoto et al., 1995; Christakou et al., 2013), schizophrenia (Martin and Albers, 1995; Joyal et al., 2004; Andreasen and Pierson, 2008), attention deficit hyperactivity disorder (Berquin et al., 1998; Mostofsky et al., 1998; Castellanos et al., 2001; Durston et al., 2011), mood swings and bipolar disorders (Strakowski et al., 2005; Baldaçara et al., 2011), and impairment in cognitive functions (Gasbarri et al., 2003).

The development of the human cerebellum begins approximately in the fourth week of gestation with the formation of the cerebellar territory in the hindbrain. Cell proliferation and migration provide the basis for further differentiation and foliation of the cerebellar surface. The occurrence of cerebellar fissures dates to the 12th week of gestation (Donkelaar et al., 2003). The human cerebellar development results in a densely folded cerebellar cortex subdivided into 10 lobules and 13 sublobules, separated by 11 fissures (Schmahmann et al., 1999) until birth (Larsell, 1947). Most importantly, within the first year of life, the human cerebellum still undergoes considerable modification. Specifically, the cortical thickness of different cerebellar subregions shows different rates of growth in neo- and archi-cerebellum, presumably depending on their origin (Tsekhmistrenko, 1996). Nevertheless, the exact dynamics of cellular and volumetric growth of different cerebellar subregions within archi- and neo-cerebellum are not fully understood. It is quite conceivable that certain cerebellar subregions, and, thus, subregion-specific cerebellar functions, develop faster than others. In any case, such potential differences in subregion-specific developmental velocity would have to be considered when investigating the developing human cerebellum in a subregion-specific manner.

**Figure 1:**
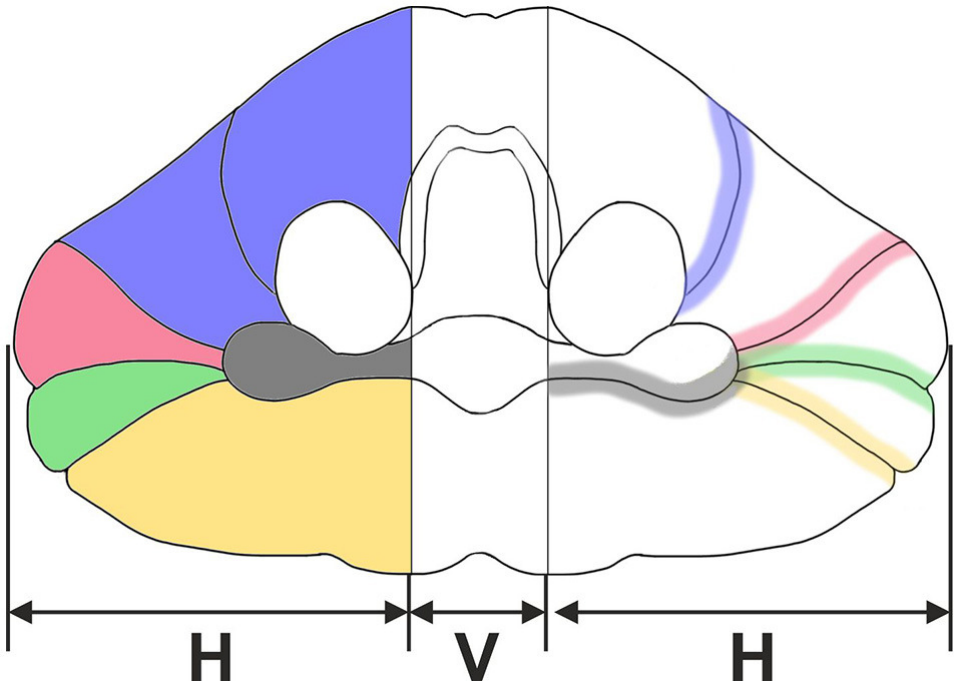
Sketch of an adult human cerebellum in ventral view. Vermis (V) and hemispheres (H) are indicated. Five regions of interest are depicted on the **left:** lobules IV-VI (blue), crus I (red), crus II (green), lobules VIIB-VIIIB (yellow), and flocculus (dark gray). Furthermore, four anatomically distinct fissures representing important landmarks on the cerebellar surface are depicted on the **right:** primary fissure (blue), superior posterior fissure (red), horizontal fissure (green), ansoparamedian fissure (yellow), and posterolateral fissure (dark gray).

Based on widely accepted macroscopic (Nieuwenhuys et al., 1980), microscopic (Skefos et al., 2014), and MRI-based (Schmahmann et al., 1999) delineations of the adult human cerebellar cortex, five different ROIs were determined in the present study for the early postnatal (<1 year of age) human cerebellum. The first ROI comprises lobules IV–VI of the cerebellar hemisphere (blue area in Figure 1) and is bordered by the superior posterior fissure (red in Figure 1). The second ROI comprises crus I (red area in Figure 1) and is bordered by the superior posterior fissure and the horizontal fissure (green in Figure 1). The third ROI comprises crus II (green area in Figure 1) and is bordered by the horizontal fissure and the ansoparamedian fissure (yellow in Figure 1). The fourth ROI comprises lobules VIIB–VIIIB (yellow area in Figure 1) and is bordered by the ansoparamedian fissure and the posterolateral fissure (dark gray in Figure 1). The fifth ROI comprises the flocculus (gray area in Figure 1).

We proposed that unequivocal identification of these ROIs is possible in the early postnatal (<1 year of age) human cerebellum, comparable to the situation in the adult human cerebellum based on the descriptions by Nieuwenhuys et al. (1980), Schmahmann et al. (1999), and Skefos et al. (2014). This was tested in the present study.

## Materials and methods

The present study was performed on post mortem cerebellar halves obtained from 20 children aged between 1 day and 11 months with known clinical records (Table 1). The cerebellar halves were collected at the Institute of Legal Medicine, Faculty of Medicine, LMU Munich (Munich, Germany) between 1999 and 2001. The mean post mortem interval (time between death and autopsy was 32.5 ± 4.8 h (mean ± standard error of the mean) (range, 7–76). The use of these autopsy cases for scientific investigations was approved by the Institutional Review Board of the University of Rostock (Rostock, Germany) under registration number A 2012-0053. Further consent to be obtained from the next of kin was not needed as per German regulations and was also waived by the ethics committee that approved the study.

**Table 1:** Characteristics of the cases investigated in the present study.

**Case no**.	**P**	**Age [m]**	**G**	**BoL [cm]**	**BoW [kg]**	**BrW [g]**	**Cause of death**	**H**
1	B	0.03	F	49	2.7	335	Suffocation	R
2	B	0.03	F	50	3.3	n.d.	Strangling	L
3	B	0.83	M	54	4.3	540	Suffocation (crime)	L
4	A	1	F	47	2.5	387	Infection	L
5	B	1.5	M	61	5.4	594	Heart defect	L
6	A	2.5	M	62	5.6	583	SIDS	L
7	B	3	M	63	5.5	721	Infection	R
8	B	3	M	55	5.4	539	WFS	L
9	A	4	F	65	6.3	723	SIDS	L
10	B	4	M	64	6.6	826	Unknown	R
11	A	6	M	69	6.7	859	SIDS	L
12	B	7	M	70	6.8	751	Suffocation (crime)	L
13	A	8	F	73	7.0	798	SIDS	R
14	B	8	M	72	9.7	1239	Otitis media	L
15	B	9	F	75	7.8	911	MCAD deficiency/AGS	L
16	A	10	F	81	11.5	1229	Suffocation (peanut)	L
17	B	10	M	74	8.9	956	Sepsis	L
18	B	10	F	65	5.5	836	Carbon monoxide intoxication	L
19	B	10	F	73	8.6	967	Myocarditis	L
20	B	11	M	73	8.8	960	Heart defect	L

*P, processing; A, processing of cerebellar halves as described in the present study; B, processing of cerebellar halves as described in Kiessling et al. (2013, 2014); m, months; G, gender; F, female; M, male; BoL, body length; BoW, body weight; BrW, brain weight; H, hemisphere; R, right; L, left; n.d., not determined; SIDS, sudden infant death syndrome; WFS, Waterhouse-Friedrichsen syndrome; MCAD, medium-chain acyl-CoA dehydrogenase; AGS, adrenogenital syndrome*.

During autopsy the cerebella were divided mediosagittally. Either the left or the right hemisphere was available for each case, and was immersion-fixed with 10% formaldehyde for 15–17 years (details are provided in Kiessling et al., 2013, 2014). Accordingly, only one hemisphere per cerebellum was investigated in the present study. Histological processing was performed at the Chair of Neuroanatomy, Institute of Anatomy, Faculty of Medicine, LMU Munich (Munich, Germany).

Six cerebellar halves (identified as A in Table 1) were rinsed in tap water for 1 week prior to being immersed in sucrose solution in Tris-buffered saline (10, 20, and 30%) at 4°C until they sank to the bottom of the jar containing the sucrose solution. Then, cerebellar halves were swabbed with paper tissue to remove fluid on the surface, meninges and vessels were carefully removed, and photographs of the cerebellar halves from different perspectives (dorsal, ventral, cranial, caudal, lateral, and medial) were taken with a Canon EOS 5D Mark III camera and Canon EF 24–105 mm 1:4.0 L IS USM objective (Canon, Tokyo, Japan).

**Figure 2:**
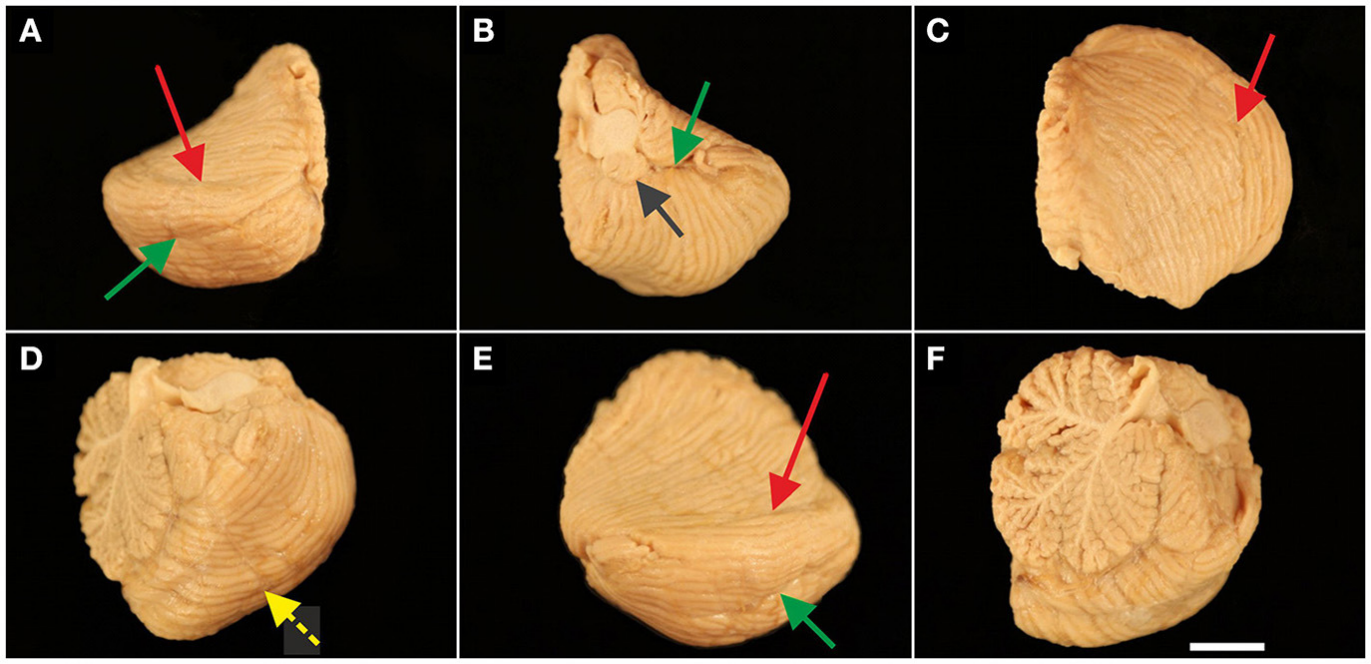
Representative left cerebellar half from a 6-month-old child (case no. 11 in Table 1). The cerebellar half is shown from dorsal **(A)**, ventral **(B)**, cranial **(C)**, caudal **(D)**, lateral **(E)**, and medial **(F)** views. The arrows indicate prominent, macroscopically visible fissures (colors of the arrows are the same as those used in Figure 1): superior posterior fissure (red arrow in **A,C,E**), horizontal fissure (green arrow in **A,B,E**), and posterolateral fissure (dark gray arrow in **B**). Identification of prominent, macroscopically visible fissures was less obvious in caudal view [the dotted yellow arrow in **(D)** points to the ansoparamedian fissure] and was not unequivocally possible in medial view. The scale bar in **(F)** represents 1 cm in **(A–F)**.

**Figure 3:**
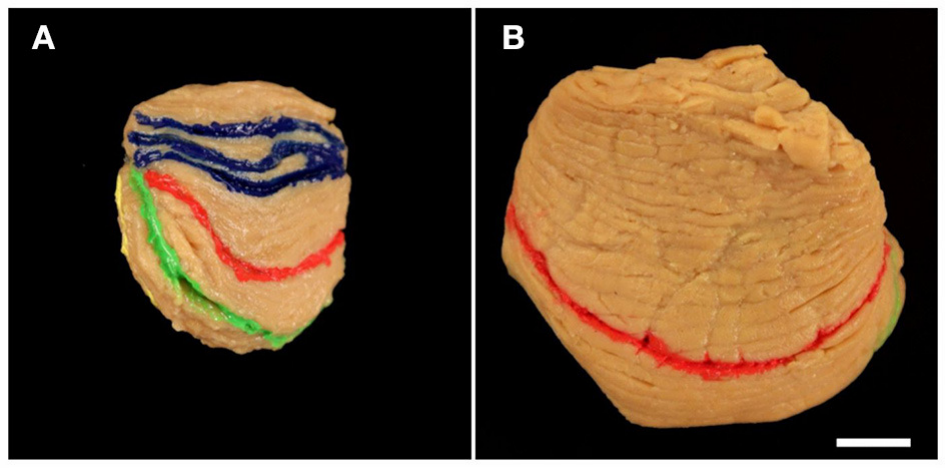
Representative right cerebellar half from a 2.5-month-old child (**A**; case no. 6 in Table 1) and left cerebellar hemisphere from a 10-month-old child (**B**; case no. 16 in Table 1) viewed from cranial. Fissures marked in blue **(A)** represent three likely positions of the primary fissure (colors are the same as those used in Figure 1). In **(B)** more than three neighboring fissures where likely to represent the primary fissure; therefore blue acrylic color was not applied. Other colored fissures are the superior posterior fissure (red in **A,B**), horizontal fissure (green in **A,B**), and ansoparamedian fissure (yellow in **A**). The scale bar in **(B)** represents 1 cm in **(A,B)**.

The surface of these six cerebellar halves were scanned to document macroscopic features including the primary, superior posterior, horizontal, ansoparamedian, and posterolateral fissures (Figure 1, right) in order to identify the following regions of interest (Figure 1, left): lobules IV–VI, crus I, crus II, lobules VIIB–VIIIB, and flocculus. Identification of fissures strictly followed the description by Schmahmann et al. (1999) based on the criteria summarized in Table 2. A representative example is shown in Figure 2. Identified fissures were filled with artist acrylics of different colors (distributed by Aldi, Mülheim an der Ruhr, Germany) mixed with tap water (3/1 v/v) (same colors as in Figure 1). In some cases, individual fissures could not be unequivocally identified and more than one fissure were filled with the same acrylic color (see Figure 3). After filling identified fissures with acrylic colors the cerebellar halves were again photographed from the same perspectives.

**Table 2:** Criteria used for identification of fissures in the early postnatal human cerebellum according to Schmahmann et al. (1999).

**Fissure**	**Description**
Horizontal	Separates lobule VIIAf from lobule VIIAt in the vermal region, and crus I from crus II in the hemispheres
Superior posterior	Separates lobule VI from lobule VII in the vermis and lobule VI from crus I (of the ansiform lobule) in the hemisphere
Posterolateral	Forms the boundary between the posterior lobe of the cerebellum and the flocculonodular lobe, separating lobule IX from lobule X (in older terminology–nodulus at the vermis; flocculus at the hemisphere)
Primary	Distinguishes the anterior lobe of the cerebellum (lobules I through V) from the posterior lobe (lobules VI through IX), and specifically it separates lobule V from lobule VI, both in the vermis and the hemisphere
Ansoparamedian	Is submerged on the ventral surface of the “tuber,” separating lobules VIIAt from VIIB (previously termed the paramedian or gracile lobule)

**Figure 4:**
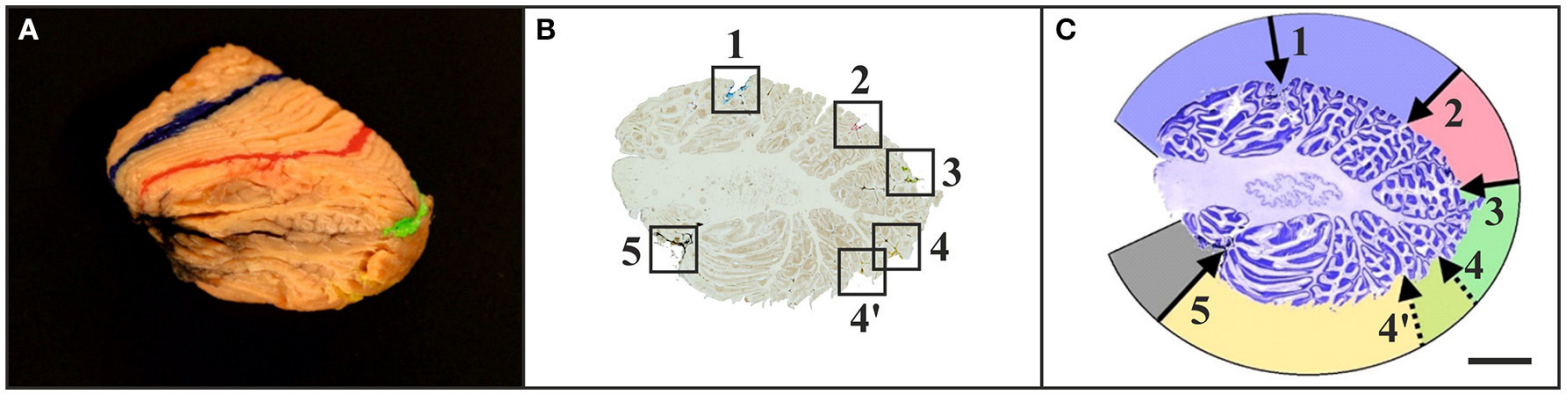
Representative left cerebellar half from a 6-month-old child (case no. 11 in Table 1) after filling identified fissures with acrylic colors **(A)** (colors as in Figure 1), cutting the cerebellar half into 100 μm-thick sagittal sections **(B)**, and staining the sections with cresyl violet **(C)**. The squares in **(B)** indicate the positions where the acrylic colors were clearly visible on unstained sections. Marking the positions of the acrylic colors by scratches on the lower surface of the glass slides facilitated recognizing of identified fissures on sections after staining with cresyl violet **(C)** [the numbers in **(C)** correspond to the numbers in **(B)**]. This allowed identification of regions of interest on histological sections of early postnatal (<1 year of age) human cerebella based on macroscopic identification of fissures representing important landmarks on the cerebellar surface. Numbers in **(B,C)**: 1, primary fissure (blue in **A,B**); 2, superior posterior fissure (red in **A,B**); 3, horizontal fissure (green in **A,B**); 4, ansoparamedian fissure (yellow in **A,B**); 5, posterolateral fissure (dark gray in **A,B**). Colors in **(C)** indicate lobules IV–VI (blue), crus I (red), crus II (green), lobules VIIB-VIIIB (yellow), and flocculus (dark gray). Note that in this example two neighboring fissures were likely to represent the ansoparamedian fissure (indicated by 4 and 4′). Therefore, both fissures were labeled with yellow acrylic color **(A,B)**, and the border between crus II and lobules VIIB-VIIIB could not unequivocally be determined **(C)**. The scale bar in **(C)** represents 1 cm in **(A**–**C)**.

Afterwards, the cerebellar halves were frozen in dry ice for 1 h and were cut into 100 μm-thick serial sagittal sections using a cryostat (Type CM 1950; Leica Microsystems, Wetzlar, Germany) equipped with C35 blades (Feather Safety Razor, Osaka, Japan). Five subsequent series of every 24th section each (with random starting points determined by a random number generator) encompassing the entire cerebellar half (distance between sections: 24 × 100 μm, which equals 2.4 mm) were collected. Four of these series of sections were stored at −20°C; one series of every 24th section per cerebellar half was randomly selected for further processing and mounted on either Superfrost plus glass slides (Menzel, Braunschweig, Germany) or gelatin-coated glass slides (Menzel). Sections were placed on a light box (Prolite Basic; Kaiser Fototechnik, Buchen, Germany) and photographed with the camera mentioned above (Figure 4). Fissures filled with acrylic colors were identified and marked by scratches on the lower surface of the glass slides. Then, sections were stained with cresyl violet, coverslipped with Malinol (Waldeck Division Chroma, Münster, Germany) and photographed again (Figure 4).

The other 14 cerebellar halves (identified as B in Table 1) had already been processed and were used in previous studies (Kiessling et al., 2013, 2014). Processing of these 14 cerebellar halves was identical to the description above except for the filling of fissures with acrylic colors and marking of the glass slides.

The final figures were assembled using Corel Photo-Paint X8 and Corel Draw X8 (both versions 18.1.0.661; Corel, Ottawa, Canada). Only minor adjustments of contrast and brightness were made, without altering the appearance of the original images.

## Results

### Identification of anatomically distinct fissures in the early postnatal human cerebellum

Tables 3, 4 summarize the findings of the present study with regard to the identification of anatomically distinct fissures in early postnatal human cerebella based on combined macroscopic and microscopic investigation. The superior posterior, horizontal, and posterolateral fissures could be unequivocally identified on all six cerebellar halves that were macroscopically investigated in the present study, irrespective of age (“A” cases in Table 1). In contrast, the primary and ansoparamedian fissures could not be reliably identified because, at the macroscopic level, they did not differ from neighboring fissures.

**Table 3:** Summary of the findings of the present study based on macroscopic and microscopic investigation.

**Fissure**	**Criteria for macroscopic investigation**	**Criteria for microscopic investigation**
	**(1) Unequivocal, reproducible identification possible**	
Horizontal	- Courses along the cerebellar equator - Dorsal: rather oblique course toward the vermis - Ventral: ending in the flocculus	- Bottom of the fissure nearby the tapering part of the white matter - Characteristic triangular shape of crus II
Superior posterior	- Prominent and uniform position at the cerebellar surface	- Proximal fissure next to the horizontal fissure in cranial direction
Posterolateral	- Unique cerebellar localization and structure, considerably differing from other cerebellar regions	- First fissure next to the cerebellar peduncle in caudal direction
	**(2) Unequivocal, reproducible identification not possible**	
Primary	- No characteristic traits compared to surrounding fissures	- Proximal fissure next to the superior posterior fissure in cranial direction
Ansoparamedian	- Morphological variability of the bordering lobules crus II and VIIB	- Proximal fissure next to the horizontal fissure in caudal direction - Fifth fissure next to the flocculus in cranial direction

**Table 4:** Summary of the findings of the present study with regard to identification of anatomically distinct fissures in early postnatal (<1 year of age) human cerebella of the cases summarized in Table 1.

**Case no**.	**P**	**Age [m]**	**H**	**PF**	**SPF**	**HF**	**APF**	**PLF**	**N-F**	**R**
1	B	0.03	R	(+)	+	+	(+)	+	11–12	
2	B	0.03	L	(+)	+	+	(+)	+	10–11	
3	B	0.83	L	(+)	+	+	(+)	+	12–13	
4	A	1	L	−	+	+	(+)	+	7–8	†
5	B	1.5	L	−	−	−	−	−	n.d	‡
6	A	2.5	L	(+)	+	+	(+)	+	9–10	‡
7	B	3	R	(+)	+	+	(+)	+	10–11	
8	B	3	L	(+)	+	+	(+)	(+)	10–11	‡
9	A	4	L	(+)	+	+	(+)	+	9–10	
10	B	4	R	(+)	+	+	(+)	+	11–12	
11	A	6	L	(+)	+	+	(+)	+	8–9	‡
12	B	7	L	−	+	+	(+)	+	9–10	‡
13	A	8	R	(+)	+	+	(+)	+	9–10	
14	B	8	L	(+)	+	+	−	+	11–12	
15	B	9	L	(+)	+	+	(+)	−	9–10	‡
16	A	10	L	(+)	+	+	(+)	+	11–12	
17	B	10	L	−	+	+	(+)	+	11–12	
18	B	10	L	(+)	+	+	(+)	+	9–10	
19	B	10	L	−	+	+	(+)	+	11–12	‡
20	B	11	L	(+)	+	+	(+)	+	10–11	

*P, Processing; A, processing of cerebellar halves as described in the present study; B, processing of cerebellar halves as described in Kiessling et al. (2013, 2014); m, months; H, hemisphere; R, right; L, left; PF, primary fissure; SPF, superior posterior fissure; HF, horizontal fissure; APF, ansoparamedian fissure; PLF, posterolateral fissure; n.d., not definable; +, unequivocal identification possible; (+), identification only approximately possible because two neighboring fissures were equally likely; −, identification not possible; N-F number of fissures; R, remarks; †, abnormal/missing fissures; ‡, minor foliation within the anterior lobe compared to the adult human cerebellum*.

**Figure 5:**
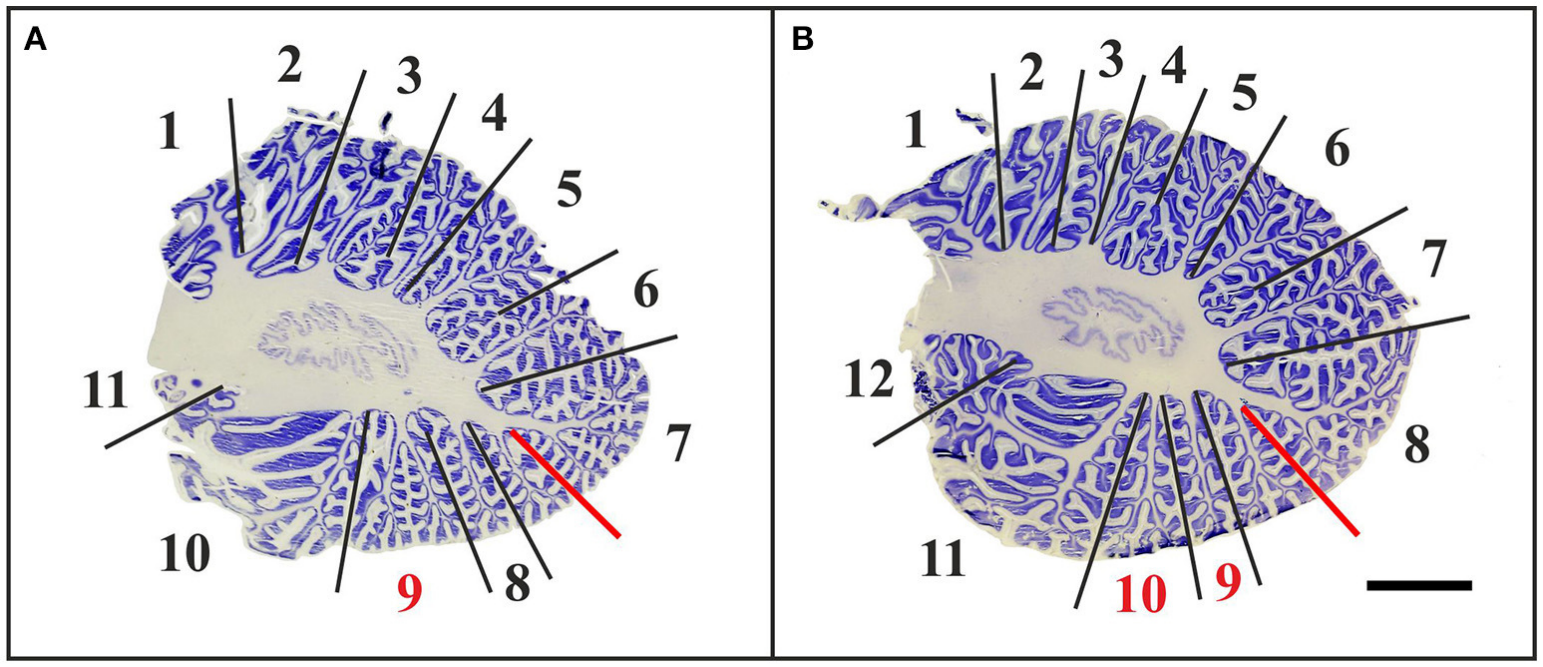
Representative 100-μm-thick parasagittal, cresyl violet-stained sections of the left cerebellar hemisphere from an 8-month-old child (**A**; case no. 14 in Table 1) compared to the left cerebellar hemisphere of a 10-month-old child (**B**; case no. 17 in Table 1). Dividing the cerebellar cortex into subregions is based on fissures reaching the central white matter (continuous lines). The red lines indicate uncertain subdivisions. When counting these in-between lobules, the total number of lobules extends to at least 11 in the cerebellum of the 8-month-old child (numbers in **A**), whereas the cerebellum of the 10-month-old child displays 12 lobules (numbers in **B**). The red numbers in **(A,B)** indicate morphologically variable regions that are part of lobule VIIIA. The scale bar in **(B)** represents 1 cm in **(A**,**B)**.

The histologic sections of five out of the six “A” cases did not allow the unequivocal identification of 10 cerebellar lobules subdivided into 13 sublobules and separated by 11 fissures. Because of a variable number of folia, the total number of lobules and sublobules differed among the cases (Figure 5). The superior posterior fissure and horizontal fissure could be readily identified on all histologic sections, but the primary and ansoparamedian fissures were not reliably identifiable. According to several references (see for example Paxinos, 1990), the primary fissure directly abuts the superior posterior fissure, and together they define the borders of crus I. However, other studies described a different localization of the primary fissure. For instance, Schmahmann et al. (1999) described that crus I includes an additional fissure. In addition, the ansoparamedian fissure was described in the literature as directly adjoining the horizontal fissure (Paxinos, 1990). However, on the histologic sections of the cerebellar halves of the “A” cases the exact position of the ansoparamedian fissure could not be identified because of slight morphologic differences in lobule VIIIA. Specifically, lobule VIIIA appeared V-shaped in cases no. 7, 12, 13, and 14 (the latter is depicted in Figure 5A) and apparently consisted of two folia. However, these two folia were not completely separated by a fissure and converged in one shared tail of white matter, which ended in the central white matter (case no. 14 in Figure 5A). In contrast, at the corresponding position on histologic sections of the other four cerebellar halves, there were two single folia, fully separated by a fissure (case no. 17 in Figure 5B).

**Figure 6:**
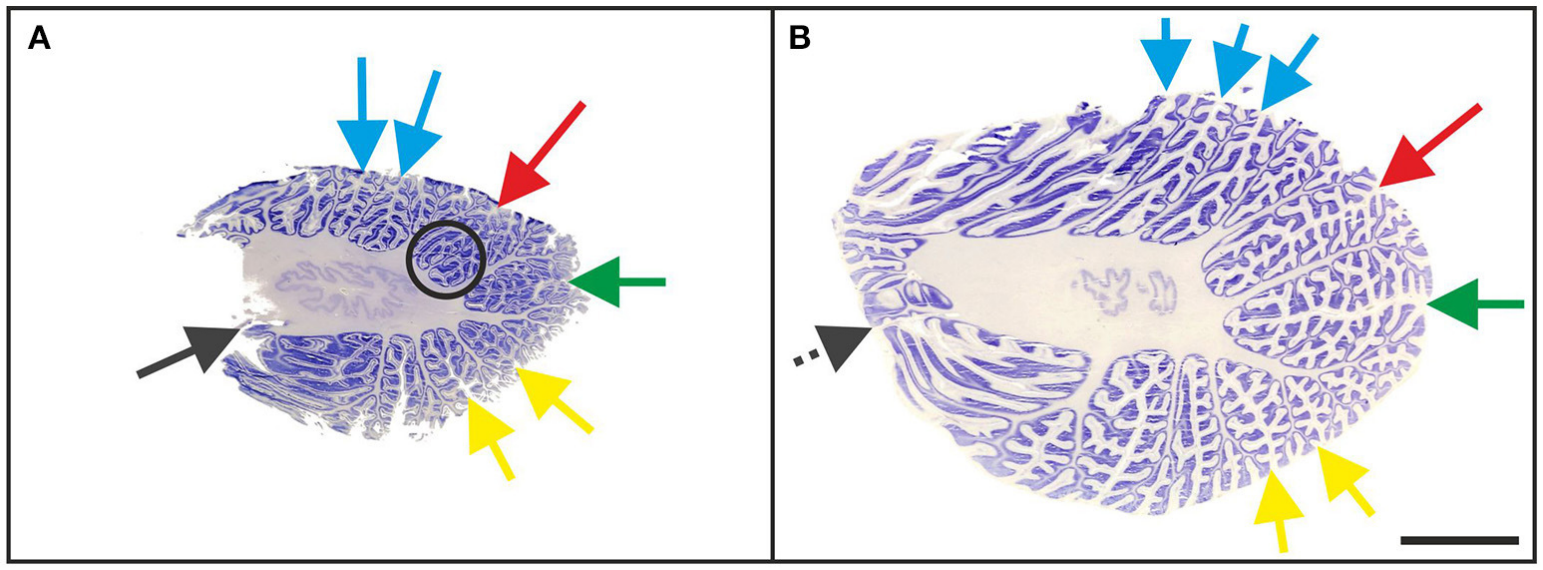
Representative 100-μm-thick parasagittal, cresyl violet-stained sections of the left cerebellar hemisphere from a 3-month-old child (**A**; case no. 7 in Table 1) and an 11-month-old child (**B**; case no. 20 in Table 1). The arrows indicate prominent visible fissures (colors as in Figure 1) as follows: primary fissure (blue), superior posterior fissure (red), horizontal fissure (green), and ansoparamedian fissure (yellow). Parasagittal sections were most suitable for microscopic identification of the posterolateral fissure (dark gray arrow in **A**), while on more lateral sections the posterolateral fissure could not be unequivocally identified because of the fusion of the flocculus with the surrounding cerebellar tissue (dotted dark gray arrow in **B**). The circled area represents accessory folia at the bottom of the superior posterior fissure. The scale bar in **(B)** represents 1 cm in **(A,B)**.

In all “A” cases, small-sized accessory folia were found at the bottom of fissures, particularly of the superior posterior fissure (Figure 6A). These folia could not be unequivocally related to the regions of interest shown in Figure 1 because of their position below the surface of the cerebellar halves.

### Histologic identification of anatomically distinct fissures in the early postnatal human cerebellum without prior macroscopic assessment

In “B” cases (Table 1), in which a macroscopic evaluation of fissures was not available, microscopic identification of the superior posterior and horizontal fissures on sagittal sections of the cerebellum was as reliable as in the “A” cases. In contrast, in two out of the 14 “B” cases the ansoparamedian fissure could only be approximated due to V-shaped areas in lobule VIIIA (as described in the “A” cases). Also, it was not possible to identify the posterolateral fissure of two “B” cases with certainty (cases no. 5 and 15). Because of the clear structure of the flocculus, the posterolateral fissure could be identified in macroscopic investigations. However, this was not possible when fissures and lobules could only be evaluated two-dimensionally at the histologic level. Specifically, it was not possible to identify the posterolateral fissure unequivocally on sections showing the transition zone from the flocculus to lobule VIIIB (Figure 6B).

As such, reproducible delineation of the regions of interest shown in Figure 1 based on isolated inspection of histologic sections was not possible because of confounding fissures that were found particularly in lateral sections. Table 3 summarizes the findings in regard to identification of anatomically distinct fissures on histologic sections of early postnatal human cerebella without macroscopic examination. This approach yielded less reliable results than when a macroscopic evaluation was conducted before histologic processing. None of these observations were restricted to either the left or the right cerebellar hemisphere.

## Discussion

### Validity of the results

The use of intact, macroscopically and microscopically well-preserved human cerebellar halves allowed for high-precision morphologic investigations such as the identification of anatomically distinct fissures and delineation of functionally relevant regions of interest. The cerebellar halves investigated in the present study represented a sample throughout the first year of life, which is a critical period of time with regard to the early postnatal development of the human cerebellum (Kiessling et al., 2014). The investigation of only one cerebellar half per case can be considered valid because biologically relevant asymmetry in the gross anatomy of the left and right halves of the human cerebellum has not been reported (Gocmen-Mas et al., 2009). Besides, the results of the present study are not influenced by the inclusion of cerebella from children who died from SIDS. Some studies postulated differences within other parts of the central nervous system between SIDS cases and matched controls. For example, Hunt et al. (2017) found altered protein expression in pontine neurons in SIDS cases. However, Kiessling et al. (2013) demonstrated using a rigorous design-based stereologic approach that there are no differences in mean volumes of the different layers within the cerebellum as well as in mean total numbers of cerebellar Purkinje cells and granule cells between SIDS cases and age- and sex-matched controls. Consequently, it is reasonable to assume that the formation and anatomical location of cerebellar fissures in SIDS cases and age- and sex-matched controls do not significantly differ from each other (as was corroborated by the findings on the “B” cases outlined in Table 4).

A potential limitation of the present study is that only cerebellar halves were investigated. As a result, the vermis was not available at full-size as it is located in-between the hemispheres, and was therefore excluded from investigation. It should however be noted that according to Schmahmann et al. (1999), there is no true “vermis” in the anterior lobe. Rather, application of this term to the paramedian sectors of the anterior lobe is an extension of the Latin term “vermis” (meaning “worm”) used by Malacarne (1776) to denote the structure visible in the posterior and inferior aspect of the cerebellum. The vermis (as such) is present from lobules VI through X. The use of the term vermis to indicate “midline” has become in time fully entrenched, and has brought with it the problem of defining what is the lateral extent of the anterior lobe “vermis” (Schmahmann et al., 1999). It has been suggested that the paravermian sulcus limits the vermis laterally. However, in many brains there is no paravermian sulcus and where one appears to be present, it may simply reflect the indentation produced by the course of the medial branch of the superior cerebellar artery (Schmahmann et al., 1999). Furthermore, the findings of the present study cannot be directly transferred to the adult human cerebellum as the definite formation of different cerebellar regions is still in process after the first year of life (Tsekhmistrenko, 1996).

Another potential issue is that the cerebellar halves investigated in the present study were not uniformly randomly sampled, e.g., by the flip of a coin, to decide whether the left or the right half of a given cerebellum was sampled and analyzed. However, the results summarized in Table 4 clearly demonstrate that the main finding of the present study (impossibility to identify reliably and reproducibly the primary and ansoparamedian fissures in the early postmortem human cerebellum) did not depend on whether the left or the right hemispheres were investigated.

### Identification of subregions of interest in the early postnatal human cerebellum

The key result of the present study was that identification of the regions of interest shown in Figure 1 based on the criteria established by Schmahmann et al. (1999) is only partially possible in the early postnatal (<1 year of age) human cerebellum. Division of the human cerebellum into five subregions of interest as outlined in Figure 1 represents a combination of functional and morphologic aspects. Indeed, the superior posterior, horizontal, and posterolateral fissures could be reliably detected on the 20 human cerebella that were investigated in the present study. In contrast, reliable and reproducible identification of the primary and ansoparamedian fissures was not possible. In this regard, Schmahmann et al. (1999) noted that the primary fissure and other fissures within the anterior lobe are progressively more difficult to discern on parasagittal sections as one moves laterally away from the midline. Specifically, the primary fissure is unmistakable on midsagittal sections but it is continuous with an undistinguished small fissure in the intermediate sectors of the hemispheres.

Accordingly, it appears feasible to perform subregion-specific investigations on early postnatal human cerebella when the identification of subregions is restricted to crus I (bordered by the superior posterior and horizontal fissures) and the flocculus (bordered by the posterolateral fissure). These subregions could be unequivocally and reliably identified on all 20 human cerebella that were investigated in the present study.

Considering the functional relevance of crus I and the flocculus, only isolated investigations of the latter would be likely to provide biologically relevant results. This is due to the fact that control of eye movements is specifically represented in the flocculonodular lobe whereas crus I cannot be functionally separated from lobulus VI and crus II (Timmann, 2012). As a consequence, isolated investigations of the flocculus would be superior to investigations of crus I taken out of context. Separate investigations of the other regions of interest shown in Figure 1 appear not possible because the associated fissures cannot be reliably identified.

The present study is the first to address whether the five regions of interest shown in Figure 1 can be reliably delineated on early postnatal human cerebella. Validity and suitability of the criteria for identification of cerebellar fissures of the early postnatal human cerebellum as summarized in Tables 2, 3 requires further investigation. These criteria were derived from reports on delineation of subregions of the adult human cerebellum using a variety of methods. Specifically, Schmahmann et al. (1999) generated an atlas of the adult human cerebellum using high-resolution MRI images. Concerning the identification of fissures, these authors mentioned that the exact delineation of the ansoparamedian fissure in the adult human cerebellum had already led to controversies in the past. It should be mentioned that Schmahmann et al. (1999) indicated the precise position of the ansoparamedian fissure on their MRI scans. On the other hand, these authors did not provide reproducible criteria for identification that could be used in design-based stereologic studies of the early postnatal human cerebellum at the microscopic level. Moreover, one cannot exclude that the discrepancies between the results of the present study and the atlas of Schmahmann et al. (1999) are at least in part due to the fact that the latter was established on MRI scans obtained from a single subject. It seems important to repeat the work of Schmahmann et al. (1999) on a larger sample, also including early postnatal (<1 year of age) subjects.

Skefos et al. (2014) determined Purkinje cell densities in the cerebellum of eight cases with autism aged 5–56 years and eight controls aged 4–52 years with design-based stereology. The authors divided the cerebellum into four different subregions, using the primary, horizontal, ansoparamedian, and posterolateral fissures as landmarks. Skefos et al. (2014) found the mean overall Purkinje cell density to be lower in the cases with autism compared to controls, with this effect being most prominent in crus I and II. As outlined above, reliable and reproducible identification of subregions of the human cerebellum as proposed by Skefos et al. (2014) was not possible in the present study. In this regard, it is worth noting that Skefos et al. (2014) did not investigate early postnatal human cerebella, and illustrated their procedure for identifying subdivisions of the human cerebellum on a single histologic section without providing the age of the corresponding subject. Accordingly, it remains unclear whether the discrepancy between the results by Skefos et al. (2014) and the results of the present study may be related to the age of the investigated subjects. In any case, we found no correlation between the age of the subjects and the number of distinct cerebellar fissures (as well as the ability to identify them unequivocally, see Table 4). It should also be mentioned that Skefos et al. (2014) stated that in some cases as much as 10% of the tissue had been lost during processing. In addition, some sections demonstrated fraying at the edge of the folia. These complications prevented Skefos et al. (2014) from estimating total numbers of Purkinje cells, and in some cases not all regions of interest could be completely sampled and analyzed.

### Relevance of design-based stereologic investigations of the entire early postnatal human cerebellum

As outlined above it is not feasible to perform design-based stereologic studies on functionally relevant subregions in the early postnatal human cerebellum, except for the flocculus and crus I. However, this does not imply that design-based stereologic studies of the early postnatal human cerebellum, with the entire cerebellum as region of interest, are of inferior significance, as in fact demonstrated by a number of studies.

Kiessling et al. (2014) investigated 14 cerebellar halves (with different causes of death other than SIDS) aged between 1 day and 11 months after birth with design-based stereology. These authors determined total numbers of cerebellar Purkinje cells and granule cells, as well as volumes of the different cerebellar layers. The total number of Purkinje cells was stable across the investigated age span, and the mean total number of Purkinje cells (13.0 × 10^6^; Kiessling et al., 2014) was similar to the mean total number of Purkinje cells in the adult human cerebellum reported in the literature: 15.3 × 10^6^ Purkinje cells were reported by Andersen et al. (1992) as well as by Korbo and Andersen (1995), 14.3 × 10^6^ by Andersen and Pakkenberg (2003) and Andersen et al. (2003), 14.9 × 10^6^ by Andersen (2004), and 11.2 × 10^6^ by Agashiwala et al. (2008). It should be noted that in all of these studies—except Kiessling et al. (2014)—estimated mean total bilateral numbers of cerebellar Purkinje cells were reported, which were divided by two in the present study to compare them to the estimated mean total unilateral number of cerebellar Purkinje cells reported by Kiessling et al. (2014). In contrast to the adult human cerebellum, total numbers of cerebellar granule cells yielded very different results. Specifically, compared to the adult human cerebellum, only ~15% of the cerebellar granule cells were found at their final position in the inner granule cell layer by the time of birth (Kiessling et al., 2014), indicating that about 85% of the final numbers of these cells are yet to be generated postnatally. These data were supported by an age-related increase of the volume of the cerebellar molecular layer, inner granule cell layer, and white matter. Kiessling et al. (2014) concluded that the human cerebellum, based on its high plasticity, might be remarkably sensitive to external and internal influences during the first year of life. In addition, these authors proposed a very high plasticity of the early human postnatal cerebellum to be related to acquisition of novel skills. In this context, Knickmeyer et al. (2008) examined the cerebellum of healthy humans in the course of the first 2 years of life with MRI, and found a volumetric increase of 240% of the cerebellum during the investigated period, suggesting that the early postnatal cerebellar growth may be directly linked to motor learning. Consistent with this hypothesis, Johnson (2001) emphasized the relevance of behavioral tests on children for a better understanding of functional brain development including the cerebellum. It should also be mentioned that Groszer et al. (2008) found abnormal cerebellar foliation and deficits in motor learning of mice carrying a mutation associated with speech impairments in humans.

Based on earlier reports in the literature that the cerebellum could be involved in the neuropathology of autism (for review see, Palmen et al., 2004), Whitney et al. (2009) investigated 10 cerebellar halves (six cases with autism and four controls aged between 17 and 54 years) with design-based stereology. Whitney et al. (2009) examined volumes of cerebellar layers and densities of cerebellar Purkinje cells, basket cells, and stellate cells. No statistically significant difference was found between the cases with autism and controls. The biological significance of these data arises from the fact that numerical matching between the cerebellar Purkinje cells and their associated interneurons provides an indication of the developmental time span for characteristic impairments related to autism. Synaptic contacts to the Purkinje cells are essential for survival of basket and stellate cells during cerebellar development, and basket and stellate cells undergo cell death if Purkinje cells are not present at the time when interneurons could establish synaptic contacts (Sotelo and Triller, 1979; Feddersen et al., 1992). Thus, in case of early Purkinje cell loss (or developmental disturbance of the formation of Purkinje cells) basket and stellate cells are also reduced in number (Whitney et al., 2009), resulting in severe defects in the development of the mouse cerebellum with impact on both foliation and size (Feddersen et al., 1992; Smeyne et al., 1995). In contrast, loss of Purkinje cells after formation of synaptic contacts with basket and stellate cells does not cause obvious malformations of the cerebellum (Feddersen et al., 1992) as Purkinje cell death does no longer affect the survival of basket and stellate cells at that time (Sotelo and Triller, 1979; Jeong et al., 2000; Duchala et al., 2004).

### Practical recommendations for design-based stereologic investigations of total numbers of cells in the early postnatal human cerebellum

#### Use human cerebella to investigate normal and pathological human cerebellar development

There are important differences in the development of the cerebellum between humans and rodents. For example, the formation of the internal granule cell layer starts prenatally in humans (Rakic and Sidman, 1970; Sidman and Rakic, 1973) but only postnatally in mice (Shimada et al., 1977; Huard et al., 1999). With regard to microvessels, capillary branching is not obvious in the rat cerebellar external granule cell layer until postnatal day 18 (Yu et al., 1994). In contrast, in the comparable stage of development in humans (1 year postnatal) branching of microvessels was observed in all cerebellar layers between the first postnatal day and 11 months of age (Müller-Starck et al., 2014). As a result, pathologic alterations of cerebellar development in mice and rats may not accurately model alterations of cerebellar development in humans. This must be considered when using animal models for research into normal and pathologic development of the cerebellum.

#### Determine the entire cerebellar cortex as ROI

It may be possible to identify reliably and delineate functionally relevant subregions in the adult human cerebellum. As outlined in the present study, this appears not to be possible in the case of the early postnatal human cerebellum, except for the flocculus and crus I. Accordingly, the entire cerebellar cortex should be determined as the ROI in order to guarantee reproducibility of results.

#### Apply different sampling schemes for counting purkinje and granule cells

At the age of 1 year there are ~2,700 times more granule cells than Purkinje cells in the human cerebellum (Kiessling et al., 2014). It is obvious that this will require two different sampling schemes, one for counting Purkinje cells and one for counting granule cells.

#### Use dynamic instead of static sampling schemes for counting purkinje and granule cells

This represents the most important difference in design-based stereologic sampling between the early postnatal and the adult human cerebellum. In the normal human cerebellum, the total number of Purkinje cells is stable during the first year of life (Kiessling et al., 2014). However, the region of interest (entire cerebellar cortex) undergoes a substantial increase in volume during this time, from ~5 cm^3^ (combined molecular layer, Purkinje cell layer, and internal granule cell layer per cerebellar half) on the first postnatal day to ~30 cm^3^ at 11 months of age (Kiessling et al., 2014). In consequence, the global Purkinje cell density (total number of Purkinje cells divided by the volume of the entire cerebellar cortex) is approximately six times higher on the first postnatal day than at 1 year of age in the human cerebellum. A static sampling scheme [i.e., an Optical Fractionator sampling scheme (West et al., 1991, 1996; Schmitz and Hof, 2005) with constant section sampling fraction and constant area sampling fraction] would not be practical to determine total number of Purkinje cells under these conditions.

Furthermore, the total number of granule cells in the internal granule cell layer increases from ~5 × 10^9^ on the first postnatal day to ~40 × 10^9^ at 11 months of age per cerebellar half (Kiessling et al., 2014). During the same time the volume of the cerebellar internal granule cell layer per cerebellar half increases from ~4 cm^3^ on the first postnatal day to ~15 cm^3^ at 11 months of age (Kiessling et al., 2014). As a result, both the global granule cell density and the total number of granule cells in the internal granule cell layer of the human cerebellum show substantial alterations during the first year of life, and a static sampling scheme would not be practical to determine total number of granule cells in the internal granule cell layer under these conditions.

Table 5 summarizes examples of proven sampling schemes for determining total numbers of Purkinje cells and granule cells in the internal granule cell layer using the Optical Fractionator method for some of the cases listed in Table 1 (taken from Kiessling et al., 2014). These exemplary sampling schemes were developed for 100-μm-thick frozen sagittal sections of the human cerebellum stained with cresyl violet (as investigated in the present study), and may serve as basis for developing reasonable dynamic sampling schemes in future design-based stereologic studies on the early postnatal human cerebellum.

**Table 5:** Examples of proven sampling schemes (taken from Kiessling et al., 2014) for determining total numbers of Purkinje cells and granule cells in the early postnatal (<1 year of age) human cerebellum using the Optical Fractionator method (West et al., 1991, 1996; Schmitz and Hof, 2005).

**CN**	**A [m]**	**∑ s**	**ssf^−1^**	**sl-g [μm]**	**sl-ucf [μm]**	**asf^−1^ [10^3^]**	**h [μm]**	**t [μm]**	**tsf^−1^**	**∑ uvcs**	**∑ n**	**CE**
**DETERMINATION OF TOTAL NUMBERS OF PURKINJE CELLS**												
1	0.03	8	36	1,300	130	0.100	25	35.4	1.42	1,435	2,377	0.021
10	4	8	48	2,800	150	0.348	45	56.0	1.24	853	720	0.037
12	7	9	48	2,900	150	0.374	45	58.7	1.30	943	744	0.037
20	11	8	48	3,100	150	0.427	45	49.7	1.14	925	573	0.042
**DETERMINATION OF TOTAL NUMBERS OF GRANULE CELLS IN THE INTERNAL GRANULE CELL LAYER**												
1	0.33	8	36	1,300	10	16.9	5	35.4	7.1	1,393	793	0.036
10	4	8	48	2,800	8	122.5	5	56.0	11.2	829	467	0.046
12	7	9	48	2,900	10	84.1	5	58.7	11.7	933	592	0.041
20	11	8	48	3,100	8	150.2	5	49.7	9.9	914	489	0.045

*CN, case number in Table 1; ∑ s, number of analyzed 100-μm thick frozen sagittal sections stained with cresyl violet; ssf^−1^, reciprocal value of the section sampling fraction; sl-g, side length in XY directions of the grid used to determine the systematic, uniformly random (SRS) positions of unbiased virtual counting spaces; sl-ucf, side length in XY directions of unbiased counting frames that serve as basis for the unbiased virtual counting spaces; asf^−1^, reciprocal value of the area sampling fraction; h, height of unbiased virtual counting spaces; t, measured actual average section thickness after histological processing; tsf^−1^, reciprocal value of the thickness sampling fraction; ∑ uvcs, number of unbiased virtual counting spaces used; ∑n, number of counted Purkinje cells or granule cells; CE, predicted coefficient of error of estimated total neuron numbers using the prediction method described by Schmitz (1998) and Schmitz and Hof (2005). Note that the sampling parameters ∑ s (and thus, ssf^−1^), sl-g, sl-ucf (and thus, asf^−1^), and h (and thus, tsf^−1^) were individually adjusted because of substantial interindividual differences in the size of the cerebella and the total number of granule cells in the internal granule cell layer (details are provided in the main text)*.

#### Consider counting other cell types in the early postnatal human cerebellum

It could be of interest also to investigate other types of cells in the early postnatal human cerebellum with design-based stereologic methods, among them the unipolar brush cells. These cells are interneurons situated in the cerebellar internal granule cell layer and the dorsal cochlear nucleus and characterized by a small (10–20 μm) soma, a single short dendritic shaft and brush-like dendritic processes (reviewed in Víg et al., 2005). They give rise to glutamatergic axons terminating on dendrites of granule cells and Golgi cell in the cerebellar glomeruli (reviewed in Mugnaini et al., 2011). The unipolar brush cells are suggested to exert feedforward amplification of single mossy fiber afferent signals that would reach the overlying Purkinje cells via ascending granule cell axons and their parallel fibers (reviewed in Mugnaini et al., 2011). These cells are intermediate in size between granule cells and Golgi cells in the mammalian cerebellar cortex (Mugnaini and Floris, 1994) which allows to distinguish them from granule cells in Nissl-stained sections. In the mouse cerebellar cortex there are at least three distinct subsets of unipolar brush cells, expressing the calcium-binding protein calretinin, the metabotropic glutamate receptor (mGluR)1α and phospholipase C (PLC) β4, and PLCβ4 but not mGluR1α (Chung et al., 2009).

So far, the unipolar brush cells in the human cerebellum were only examined in a few studies (Víg et al., 2005; Wegiel et al., 2013). Notably, Víg et al. (2005) found that in human, calretinin-immunoreactive unipolar brush cells are present in the cerebellar vermis at birth and their number increases at least until the first postnatal year. This is in line with the finding of Kiessling et al. (2014) that ~85% of the cerebellar granule cells are generated postnatally in human. Furthermore, Wegiel et al. (2013) reported a potential role of the unipolar brush cells in the neuropathology of autism.

However, neither the present study nor the previous studies by Kiessling et al. (2013, 2014) addressed the unipolar brush cells. Accordingly, the data provided in Table 5 cannot be used to develop sampling schemes for determining total number of unipolar brush cells in the early postnatal human cerebellum using the Optical Fractionator. Furthermore, neither Víg et al. (2005) nor Wegiel et al. (2013) investigated total number or density of the unipolar brush cells with stereologic methods. Accordingly, investigating these cells in the early postnatal human cerebellum would require first a pilot study to assess their regional density (note that the data provided by Víg et al., 2005 and Wegiel et al., 2013 are not sufficient in this regard), followed by a stereologic pilot study to determine their age-dependent total number. With that information, detailed stereologic studies of the unipolar brush cells and their potential role in the neuropathology of neurodevelopmental, neuropsychiatric, and neurodegenerative disorders could then be performed.

## Conclusion

Design-based stereologic studies of the early postnatal (<1 year of age) human cerebellum are required to understand better its normal and pathologic development and its role in various neurodevelopmental disorders. However, unlike for the adult human cerebellum, it is not feasible to identify reliably and delineate functionally relevant subregions in the early postnatal cerebellum, except for the flocculus and crus I. Accordingly, it is recommended to define the entire cerebellar cortex as the ROI in design-based stereologic studies and use of dynamic rather than static sampling schemes to guarantee reproducibility and reliability of results. Beyond this, it seems important to repeat the work by Schmahmann et al. (1999) who developed a MRI atlas of the human cerebellum in proportional stereotaxic space on a larger sample than a single specimen, including early postnatal subjects.

## Author contributions

AF, AB, PH, CS, and MK: Made substantial contributions to the conception and design of the work, and to the acquisition, analysis, and interpretation of data for the work; AF, AB, PH, CS, and MK: Drafted the work, approved the final version to be published, and agreed to be accountable for all aspects of the work in ensuring that questions related to the accuracy or integrity of any part of the work are appropriately investigated and resolved.

### Conflict of interest statement

The authors declare that the research was conducted in the absence of any commercial or financial relationships that could be construed as a potential conflict of interest.
